# Targeting Acyl Homoserine Lactones (AHLs) by the quorum quenching bacterial strains to control biofilm formation in *Pseudomonas aeruginosa*

**DOI:** 10.1016/j.sjbs.2021.10.064

**Published:** 2021-10-30

**Authors:** Syeda Javariya Khalid, Quratul Ain, Sher Jamal Khan, Amna Jalil, Muhammad Faisal Siddiqui, Tahir Ahmad, Malik Badshah, Fazal Adnan

**Affiliations:** a Atta ur Rahman School of Applied Biosciences (ASAB), National University of Sciences and Technology (NUST), Islamabad, Pakistan; b Institute of Environmental Sciences and Engineering (IESE), National University of Sciences and Technology (NUST), Islamabad, Pakistan; c Department of Microbiology, Hazara University, Mansehra, KP, Pakistan; d Department of Microbiology, Quaid-i-azam University, Islamabad, Pakistan

**Keywords:** Quorum sensing, Quorum quenching, Lactonase, Acylase, *N-*acyl homoserine lactones (AHL), antibiotic resistance

## Abstract

Navigating novel biological strategies to mitigate bacterial biofilms have great worth to combat bacterial infections. Bacterial infections caused by the biofilm forming bacteria are 1000 times more resistant to antibiotics than the planktonic bacteria. Among the known bacterial infections, more than 70% involve biofilms which severely complicates treatment options. Biofilm formation is mainly regulated by the Quorum sensing (QS) mechanism. Interference with the QS system by the quorum quenching (QQ) enzyme is a potent strategy to mitigate biofilm. In this study, bacterial strains with QQ activity were identified and their anti-biofilm potential was investigated against the Multidrug Resistant (MDR) *Pseudomonas aeruginosa*. A *Chromobacterium violaceum* CV026 and *Agrobacterium tumefaciens* A136-based bioassays were used to confirm the degradation of different Acyl Homoserine Lactones (AHLs) by QQ isolates. The *16S rRNA* gene sequencing of the isolated strains identified them as *Bacillus cereus* strain QSP03, *B. subtilis* strain QSP10, *Pseudomonas putida* strain QQ3 and *P. aeruginosa* strain QSP01. Biofilm mitigation potential of QQ isolates was tested against MDR *P. aeruginosa* and the results suggested that 50% biofilm reduction was observed by QQ3 and QSP01 strains, and around 60% reduction by QSP10 and QSP03 bacterial isolates. The presence of AHL degrading enzymes, lactonases and acylases, was confirmed by PCR based screening and sequencing of the already annotated genes *aiiA*, *pvdQ* and *quiP.* Altogether, these results exhibit that QQ bacterial strains or their products could be useful to control biofilm formation in *P.aeruginosa.*

## 1 Introduction

Antibiotic resistance is growing more rapidly and spreading faster than ever before (Chioro et al., 2015, Abdula et al., 2016). A report commissioned by the British government estimated that by 2050, antimicrobial resistance could cause over 10 million deaths each year and result in cumulative losses of US$ 100 trillion to world GDP (Ma et al., 2019, O’Niel, 2016, Whiteley et al., 2017). Excessive use of antibiotics create the selection pressure on microorganisms; thus, bringing on the evolution of microbial resistance (Laxminarayan et al., 2013, Zhao et al., 2017a, Zhao et al., 2017b, Liao et al., 2019). Bacterial infections caused by the biofilm forming bacteria are 1,000 times more resistant to antibiotics than the planktonic bacterial strains (Rasmussen and Givskov, 2006). More than 70% bacterial infections involve biofilms which severely complicates treatment options for most of the infections (Musk and Hergenrother, 2006). Some studies suggest that the Quorum Sensing (QS) system may also be associated with the bacterial resistance (Haque et al., 2018, Jiang et al., 2019, Zhao et al., 2017a, Zhao et al., 2017b). Quorum Sensing (QS) is basically cell to cell communication system of microorganisms that is achieved through signaling molecules collectively known as auto-inducers. They regulate and monitor changes in population density and trigger the expression of virulent genes and other infection related phenotypes in response to signals which are mostly small molecules such as oligopeptides, fatty acid derivatives or furanones (Zhang and Dong, 2004). A large number of biologically important functions such as antibiotic production, motility, luminescence, plasmid transfer, biofilm formation, virulence factors and many others are controlled by quorum sensing (Galloway et al., 2011, Whitehead et al., 2001).

Broadly, auto-inducers produced by bacteria are characterized into three types based on the differences in their chemical structure and mechanism of action: (i) Auto-inducing peptides (AIPs), (ii) Acylhomoserine lactones (AHLs), (iii) Auto-inducer 2 (AI-2) (Huang et al., 2016). Acylhomoserine lactones (AHLs)-based QS system is one of the best described communication processes of bacteria involving production, secretion and recognition of AHL auto-inducers. This type of QS signals is found in more than 70 species of bacteria, most of them are known pathogens (Williams et al., 2007, Withers et al., 2001). Any strategy to suppress their production could be promising in dealing with life threatening ailments. Several approaches to disrupt this communication process are collectively known as Quorum Quenching (Ozer et al., 2005, Hentzer and Givskov, 2003).

There are so many ways to interfere with QS mechanism. For example, many natural substances can imitate AHLs and block their recognition by receptors. Such receptors generally block the protein that works as AHL receptor and initiates the expression of target genes (Manefield et al., 1999). Some higher plants also produce metabolites that disrupt QS system in microorganisms. Some plants secret the compounds from their roots that mimic bacterial signaling molecules affecting the gene expression pattern of Rhizosphere bacteria (Gao et al., 2003). Inhibitory effect on *P*. *aeruginosa* QS system and suppression of virulence factors by plant extracts has been reported too (Adonizio et al., 2008). Synthetic QS inhibitors have also been reported (Reverchon et al., 2002).

Any factor that disrupts the QS system in bacteria has good potential to be used as a treatment strategy. However, AHL degradation achieved through enzymes produced by many bacteria described as ‘Quorum Quenching bacteria‘, is one of the most potent strategy for QS inhibition. QQ enzymes are divided into two classes on the basis of their mode of AHL degradation (Dong and Zhang, 2005): Class I includes (i) AHL lactonases which inactivates AHL by hydrolysis of lactone ring, the gene *aiiA* encoding AHL lactonase was first time reported in *Bacillus* sp. Strain 240B1. It was successfully cloned in *Escherichia coli* to study its effect on AHL substrate. When expressed in the transformed pathogen *Erwinia carotovora* strain SCG1, it successfully decreased its pathogenicity for Chinese cabbage, cauliflower, tobacco, etc. (Dong et al., 2000), and (ii) AHL acylase that cleaves the bond between fatty acids and homoserinelactone (Lin et al., 2003). This enzyme was first time discovered in *Variovorax paradoxus* strain VAI-C showing enhanced level of AHL degradation (Leadbetter and Greenberg, 2000). Whereas, class II includes oxidoreductases that bring changes in chemical structure of AHLs instead of degrading them. This causes the change in activation state of signaling molecule (Chan et al., 2011). Generally, redox reaction reduces the activity, while hydrolysis of AHL molecules results in complete loss of the activity (Chowdhary et al., 2007). This is how acylases and lactonases are more useful and potent in QQ activities.

So, quenching the quorum of bacteria has the potential to obstruct the pathogen s ability to synchronize its cell population and virulence factors. It ensures the time for host to combat infection naturally through immune system (Chen et al., 2013). Quorum Quenching (QQ) is gaining importance as a novel method to control bacterial biofilms in medical and industrial sectors, wastewater treatment plants, and aquacultures (Torres et al., 2016, Bzdrenga et al., 2017). In this study, QQ bacteria were isolated from sludge samples of membrane bioreactor that can degrade AHLs and thus interfere with the communication of bacteria. For the isolation of bacteria, sludge from the membrane bioreactor was used. This niche might help in exploring rare bacterial species that can be used for biofilm control not only in health sector but also in controlling biofouling on membrane bioreactors (MBR). Sequencing of the strains was done to identify the genes that encodes QQ enzymes. AHL degradation ability of the isolates was investigated and the QQ isolates were further evaluated for their biofilm inhibitory activity against Multiple Drug Resistant (MDR) *Pseudomonas aeruginosa*.

## 2 Methodology

### 2.1 Sludge sample collection and processing

Sludge samples were collected from a full-scale membrane bioreactor situated at H-12 Campus of National University of Science and Technology (NUST) Islamabad, Pakistan. The samples were collected from three different points of bioreactor; biotank, membrane tank, and sludge wastage point. The samples were collected in glass bottles, mixed thoroughly, and processed within an hour for subsequent bacterial isolation. Five milliliters of sludge sample was mixed with 50 ml saline, sonicated for 30 sec and centrifuged at 3000 rpm for 1 min for debris removal. Supernatant was centrifuged again at 4500 rpm for 5 min. Pellet was re-suspended in 15 ml saline and used for the isolation of QQ bacteria.

### 2.2 Isolation and characterization of QQ bacteria

The processed sludge solution was enriched with AHLs mixture (Table 1) in 3 enrichment cycles. For first^t^ enrichment cycle, 1:1 solution of processed sludge and minimal media was mixed with 1% AHLs mixture and the tubes were incubated at 37 °C for three days. For second cycle, 100 µl of the culture from first cycle was mixed with 1 ml fresh minimal media having AHLs mixture and incubated for 3 more days. The third enrichment cycle was done in the similar fashion. After the 3rd cycle, 100 µl of the culture was spread on LB agar plate and incubated at 37 °C for 48 h. Strain selection was done from the limited number of colonies appeared, based on the differences in macroscopic characteristics. Pure strains were then subjected to gram staining, biochemical testing, and growth on selective and differential media.

**Table 1:** AHLs mixture used during the enrichment process for the isolation of QQ bacteria.

S. No	AHLs Mixture
1.	N- (3-oxo-hexanoyl)-L-homoserine lactone (3OC_6_HSL) standard of 0.5 mg/ml
2.	N-(3-oxo-dodecanoyl)-L-homoserine lactone (3OC_12_HSL) standard of 0.5 mg/ml
3.	N-(3-Hydroxybutanoyl)-L-homoserine lactone
4.	N-(3-Oxodecanoyl)-L-homoserine lactone

### 2.3 Strain identification and phylogenetic analysis

For strains identification, the strains were streaked on the agar slants in cryovials and send to Macrogen Korea (Seoul Rep. of Korea) for 16S rRNA sequencing. A total of 4, 16S rRNA gene sequences were identified and compared by Blast-search (GenBank; http://www.ncbi.nlm.nih.gov). All the sequences were aligned using CLUSTAL W (Thompson et al., 1994). To understand the evolutionary relationships of the identified strains, phylogenetic analysis of each strain was performed in the form of phylogenetic tree that was constructed by Mega 7 using neighbor joining method with a bootstrap value of 100. The 16S rRNA gene sequences of *Pseudomonas aeruginosa* strain QSP01, *Bacillus cereus* strain QSP03, *Bacillus subtilis* strain QSP10, and *Pseudomonas putida* strain QQ3 were deposited in the GenBank database under the accession numbers KY576793, KY576795, KY576801, and KR058848, respectively.

### 2.4 Screening of AHL degradation activity

Four different AHLs (Sigma-Aldrich, Spain) were used: C4-HSL (N-butanoyl-L-homoserine Lactone), C6-HSL (N-hexanoyl-DL-homoserine lactone), C10-HSL (N-Decanoyl-L-homoserine lactone), and C12-HSL (N-dodecanoyl-L-Homoserine lactone). The isolated bacterial strains were analyzed for QQ activity by using the two AHL biosensor strains *Chromobacterium violaceum* CV026 for the detection of short chain AHLs (McClean et al., 1997, Romero et al., 2014, Torres et al., 2013), and *Agrobacterium tumefaciens* A136 along with X-gal, for the detection of long chain AHLs. Each strain’s AHL degradation activity was tested by two ways; the cell free lysate assay, and the whole cell assay. The whole-cell assay was performed by agar overlay method and disk diffusion method.

**Agar overlay method** was performed as described previously in literature with some modifications (Lade et al., 2014). For short chain AHLs degradation, CV026 indicator plates were prepared by growing it overnight in the presence of 20 µg/ml of Kanamycin and poured on the surface of already prepared LB agar plates. Around 6 mm wells were prepared in plates and 50 µl of each QQ bacterial strain along with 5 µM of C4-HSL, or C6-HSL was poured in those wells. Control well only contain exogenous AHLs. Plates were incubated for 48 h at 28 °C. Color in surrounding areas of well was observed. For long chain AHLs by QQ isolates, A136 indicator plates were prepared by growing it overnight in the presence of 50 µg/ml of Spectinomycin and 4.5 µg/ml of Tetracycline. Rest of the method was same as described above except in this case C10-HSL, or C12-HSL was used instead of short chain AHLs.

AHL degradation assay by Disk Diffusion method was performed as described previously with some modification (Chan et al., 2011, Chong et al., 2012). A sterile membrane filter disk was dipped in 10 mg/ml of C4-HSL, or C6-HSL and placed over the surface of CV026 indicator plate. Similarly, disk was dipped in 10 mg/ml of C10-HSL, or C12-HSL and placed over the surface of A136 plate. Fresh colonies of each QQ isolate were loaded on membrane filter disks and plates were incubated for 48 h at 28 °C and color on the plates was checked. For control, discs only contain AHLs and no QQ isolate.

The cell free lysate assay was performed for the quantification of degraded AHLs (Zhu et al., 2011). For that purpose, cell free lysate of each QQ bacterial isolate was prepared as described previously (Christiaen et al., 2011). For AHL degradation, 50 µl of CV026 culture was inoculated in LB medium supplemented with 2 µl of 0.5 mg/ml of C4-HSL and 100 µl cell free lysate and incubated at 30 °C for 24 h at 120 rpm. Then, 1:1 culture and dimethyl sulphoxide was centrifuged at 8000 rpm for 5 min to solubilize violacein and for cell removal. Supernatant was added to wells of sterile polyethylene 96-wells microtiter plate. Harvested bacterial cells were re-suspended in sterile distilled water and transferred to the wells of microtiter plate. Plates were incubated at 30 °C for 48 h and absorbance for violacein was analyzed at 550 nm, and for cell growth at 630 nm. The results were compared with assay controls which contained CV026 supplemented with only C4-HSL.

### 2.5 Biofilm inhibition test by QQ bacteria

To evaluate the biofilm inhibition ability of QQ bacterial strains, *P. aeruginosa* strain was used (Cady et al., 2012). For this purpose, 1:100 dilution of *P. aeruginosa* and TSB was prepared, 180 µl of this solution along with 20 µl cell free lysate of QQ bacterial isolate was transferred individually into the wells of 96-well microtiter plate and incubated aerobically at 37 °C for 48 h. After incubation time, optical density (OD) of planktonic cell was evaluated at 630 nm. Planktonic cells were then removed, and microtiter plate was washed to remove loosely attached cells. Tightly bound cells were then fixed with methanol, stained with crystal violet (0.1%), dissolved in glacial acetic acid (33%), and quantified at 550 nm in a microtiter plate reader. The control used was diluted bacterial culture without cell free lysate. To confirm the involvement of QQ enzymes in biofilm inhibition, exogenous C4-HSL (10 µl) was added along with cell free lysates. C4-HSL plays a significant role in biofilm formation of *P. aeruginosa* (Favre-Bonte et al., 2003). Rest of the protocol was same as mentioned above.

### 2.6 Antibiotic susceptibility testing of QQ bacteria

Isolated strains were tested for their antibiotic susceptibility pattern as the antibiotic resistance is indirectly linked with pathogenic potential, the method used was Kirby–Bauer method (Barry and Thornsberry, 1985). The antibiotics were procured from Oxoid™, UK and included; ampicillin (AMP, 10 μg), amoxicillin (AML, 10 μg), cefepime (FEP, 30 μg), gentamicin (CN, 10 μg), ceftazidime (CAZ, 30 μg), chloramphenicol (CIP, 5 μg), cephazolin (KZ, 30 μg), and ampicillin/sulbactam (SAM, 20 μg). The diameters of zones of inhibition were measured in millimeters and evaluated as Susceptible (S), Intermediate (I), or Resistant (R) according to the CLSI (CLSI, 2012). The interpretation of antibiotic susceptibility was subjected to generate a heat map depicting the hierarchical clustering of each isolate in g-plot package of R (R studio).

### 2.7 Identification of the quorum quenching genes

Well reported QQ genes were confirmed to understand the molecular basis of AHL degradation in QQ bacterial isolates. DNA of bacterial isolates was extracted through GeneJET Genomic DNA Purification Kit (Thermo Scientific™). Primers for two main categories of quorum quenching genes (Lactonase and Acylase) were designed against the conserved sequences of selected genes (Table 2). PCR amplification conditions were as follows: initial denaturation step at 95 °C for 5 min; 30 cycles of denaturation at 95 °C for 30 s, annealing at 59.9 °C for *aiiA* gene, and 62.3 °C for *pvdQ* and *quiP* genes for 40 s, extension at 72 °C for 1 min, followed by a final extension step at 72 °C for 10 min (see Table 3).

**Table 2:** Primers for two main categories of quorum quenching genes (Lactonase and Acylase).

Sr. No	Gene	Function	Primers 5′-3′		Amplicon Size (bp)	Length (bp)	GC (%)	Reference
1.	***aiiA***	AHL lactonase	F	GATGGCCTGGAGAATGAC	257	18	56	This study
			R	GCGTGTAGGGTATGAGCC		18	61	
2.	***pvdQ***	AHL acylase	F	GTTCTGCACGAAGTCCCTG	1411	19	58	This study
			R	GCTGTTGGGTTCGATGATG		19	53	
3.	***quiP***	AHL acylase	F	GTCGGCCAGGTAATAGAGC	572	19	58	This study
			R	GCTACCGTCCGGAATACTG		19	58

**Table 3:** Characterization of QQ bacterial isolates on the basis of gram staining, biochemical testing, and growth on selective and differential media.

Bacterial Isolates	QSP01	QSP03	QSP10	QQ3
Gram Staining	−ve	+ve	+ve	−ve
Morphology	Rods	Rods	Rods	Rods
Pigmentation	Off white colonies	Off white colonies	Off white colonies	Off white colonies
Catalase Test	+ve	+ve	+ve	+ve
Oxidase Test	+ve	−ve	+ve	+ve
Growth on EMB Agar	No Lactose Fermentation	No Growth	No Growth	No Growth
Growth on MacConkey Agar	Pale Colonies, no lactose fermentation	No Growth	No Growth	Pale colonies, no lactose fermentation
Growth on Mannitol Salt Agar	No Growth	Negligible growth	Negligible growth	No Growth
Growth on Cetrimide Agar	blue-green growth	No Growth	No Growth	Off white colonies

### 2.8 PCR product purification and sequence analysis

Amplified PCR product was purified by GeneJET PCR purification kit (Thermo Scientific™). The final eluted product was sent to Macrogen, Korea (Seoul Rep. of Korea) for sequencing. Consensus sequence of each sequenced gene was determined using BioEdit tool and subjected to Blast analysis. Annotated sequences of genes *aiiA, pvdQ*, and *quiP* were submitted to GenBank under the accession number MG213739, MG356411, and MG356412, respectively. ExPASy tool was used to retrieve the amino acid sequences, and Conserved domains in sequenced amplicons were determined by Conserved Domain Database (CDD) available at NCBI. Phylogenetic tree showing the evolutionary relationship of sequenced amplicons with other published data of QQ genes was built using MEGA 7 with bootstrap value of 100.

### 2.9 Statistical analysis

**Fig. 1:**
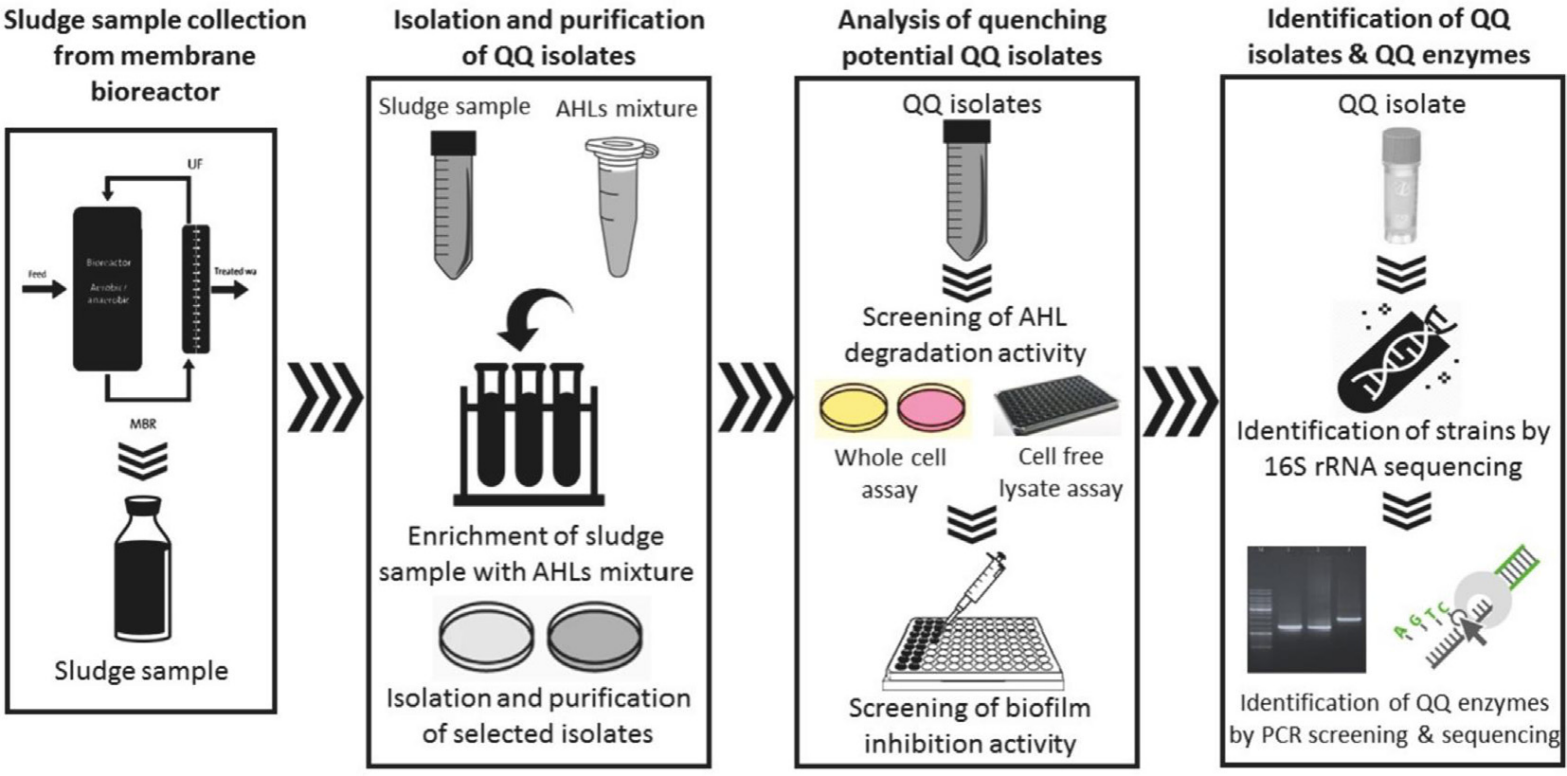
Brief sketch of the methodology adopted.

Statistical significance of the QQ effect on biofilm formation and AHL degradation quantification data was inspected by one-way ANOVA with a p value <0.05 followed by Tukeys test. Graphpad Prism 7 was used to perform these statistical analyses (see Fig. 1).

## 3 Results

### 3.1 Bacterial isolates successfully degraded AHLs

**Fig. 2:**
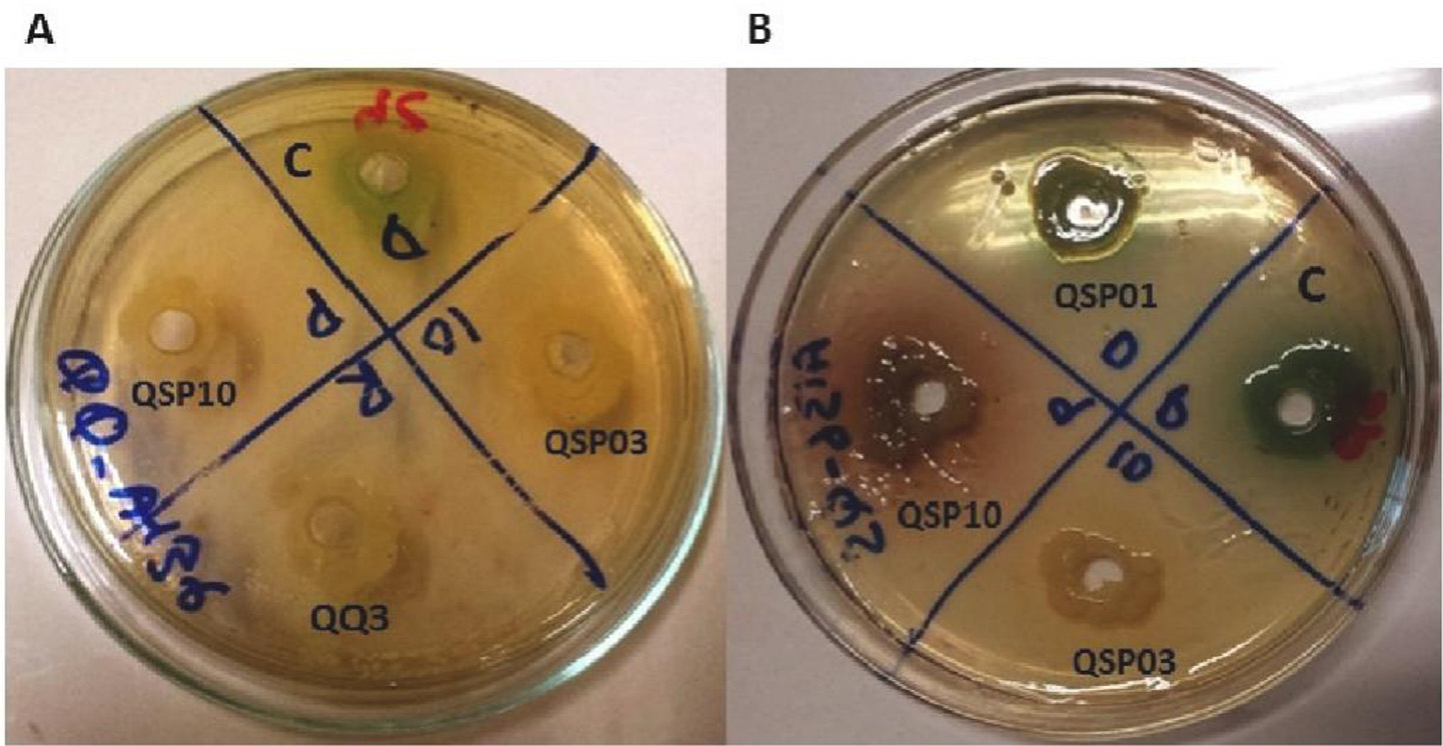
AHL degradation analysis indicating agar plates of CV026 and A136 strains. (A) A136 indicator plate with C10-HSL, control well is showing blue-green color, while no color is produced in any other well. (B) A136 indicator plate with C12-HSL, control well is showing blue-green color, while no color is produced around QSP03 well.

The four selected bacterial isolates (QSP03, QSP10, QSP01 and QQ3) were screened for their QQ activity. An agar well diffusion assay was performed using the biosensor strain *C. violaceum* CV026, that produces a violet pigment called violacein in response to short chain AHLs (in this case; C4-HSL, and C6-HSL). The Quorum Quenching isolates can degrade AHLs, so they do not allow the development of any color. Color in surrounding areas of wells was observed. Color around the well containing only C6-HSL or C4-HSL was changed from light yellow to violet (Fig. 2). It confirms the restoration of quorum sensing in CV026 in the surrounding areas of well because of exogenous AHL. Color around the wells containing bacterial isolates did not change much despite the presence of C4-HSL or C6-HSL. This confirms these bacterial isolates exhibiting quorum quenching properties as they degraded the provided AHL. All the bacterial isolates effectively degrade short chain AHLs (Table 4)

**Table 4:** QQ activity of bacterial isolates to four AHLs with various acyl chain lengths.

AHLs	Biosensor	QSP01	QSP03	QSP10	QQ3
C4-HSL (BHL)	CV026	+++	++++	++++	+++
C6-HSL (HHL)	CV026	+++	++++	++++	+++
C10-HSL (DHL)	A136	++	+++	++++	+++
C12-HSL (DdHL)	A136	++	+++	++	+++

More Significant: ++++; Significant: +++; Moderate: ++; No degradation: -

The process was repeated on A136 indicating agar plate for long chain AHL detection. C10-HSL, and C12-HSL were added as exogenous long chain AHL source. Color in surrounding areas of wells was observed. Color surrounding the well containing only exogenous AHL was changed from light yellow to blue-green. AHL here worked as signaling molecule and induced the expression of β -galactosidase gene in A136. When the enzyme acted upon its substrate X-gal, it produced greenish color. Color around the wells containing bacterial isolates did not change despite the presence of AHL. It confirms the bacterial isolates to be quorum quenching and capable of degrading long chain AHL too (Fig. 2). The same results were obtained in case of disc diffusion method (Table 4).

**Fig. 3:**
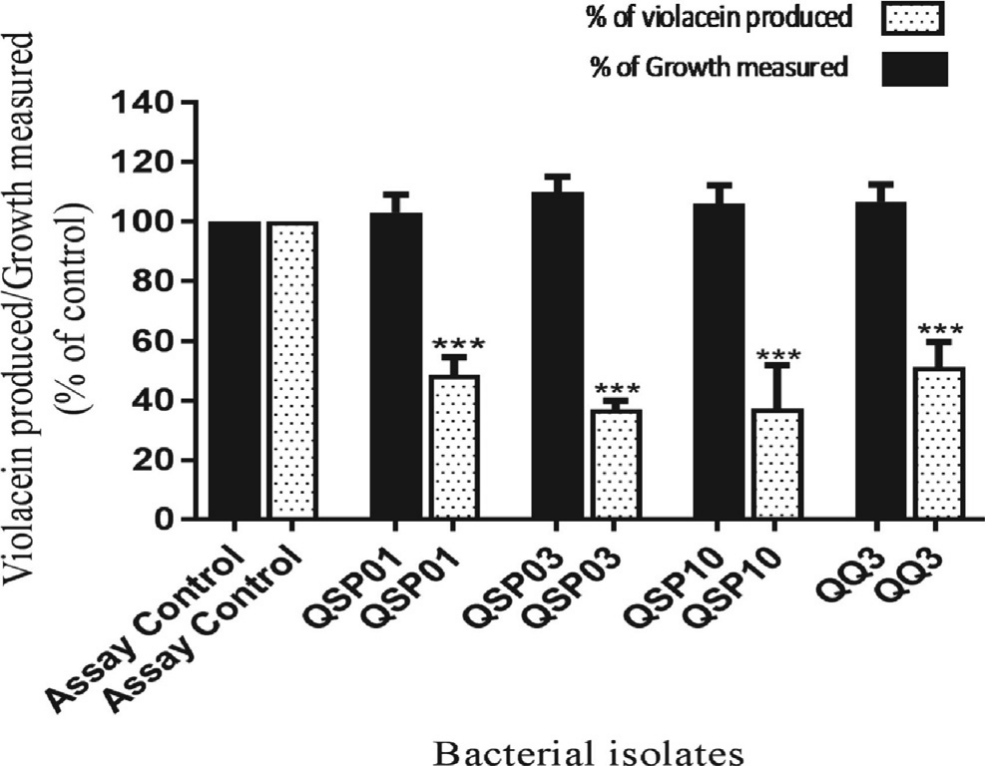
Violacein estimation by AHL degradation assay. X-axis represents CV026 culture treated with QSP01, QSP03, QSP10 and QQ3 isolates and supplemented with C4-HSL. Assay control involves CV026 culture supplemented with C4-HSL with no AHL degrading agent treatment. Y-axis shows the intensity of violacein produced and growth measured in treated and untreated cultures.

The cell free lysate assay for AHL degradation was performed for the quantification of degraded AHLs. Amount of violacein produced by CV026 is inversely related to C4-HSL degradation by QQ isolates. Each bacterial isolate inhibited violacein production to different extents. Around 60% violacein inhibition was seen by QSP03 and QSP10 whereas 50% violacein inhibition was observed by QSP01 and QQ3 strains (p > 0.0001) (Fig. 3)

### 3.2 Biofilm formation in *P. aeruginosa* was inhibited significantly by the bacterial isolates

**Fig. 4:**
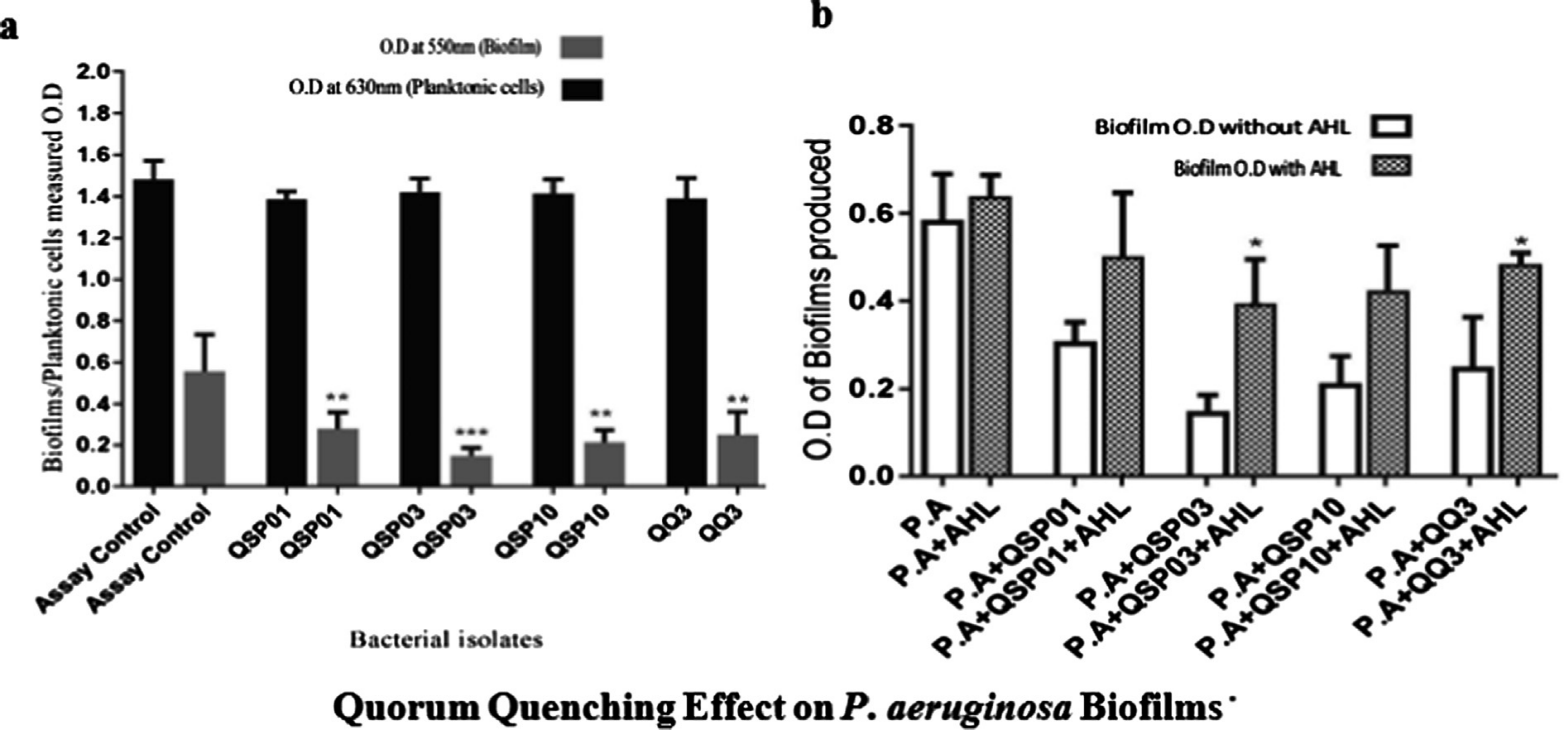
a) Biofilm degradation assay. Quorum quenching effect of QQ isolates on P. aeruginosa biofilm was analyzed by static microtiter plate method. X-axis represents P. aeruginosa cells treated with QSP01, QSP03, QSP10 and QQ3 isolates. Assay control involves P. aeruginosa without any QQ agent. Y-axis represent the planktonic cell growth and adherent cells (Biofilm) persistence in both treated and untreated cultures. b) Analyzing role of QS in biofilm formation. Biofilm formation in P. aeruginosa with and without exogenous supply of AHL. X-axis represents the biofilm formation in P. aeruginosa with and without treatment of bacterial isolates and C4-HSL. Y-axis represents the concentration of produced biofilms at 550 nm.

Significant biofilm inhibition effect was observed for each QQ bacterial isolate‘s cell free lysate treatment. About 50% biofilm inhibition was observed by QQ3 and QSP01, and almost 60% inhibition by QSP10 and QSP03 bacterial isolates (p < 0.01). Growth inhibition was not seen in case of planktonic cells. When exogenous AHL was added, the results showed significant regeneration of *P. aeruginosa* biofilms It confirms that *P. aeruginosa* biofilm inhibition by QQ bacterial lysate treated cells is due to AHL degradation effect (Fig. 4).

### 3.3 Phylogenetic analysis of the QQ isolates confirmed its lineage

**Fig. 5:**
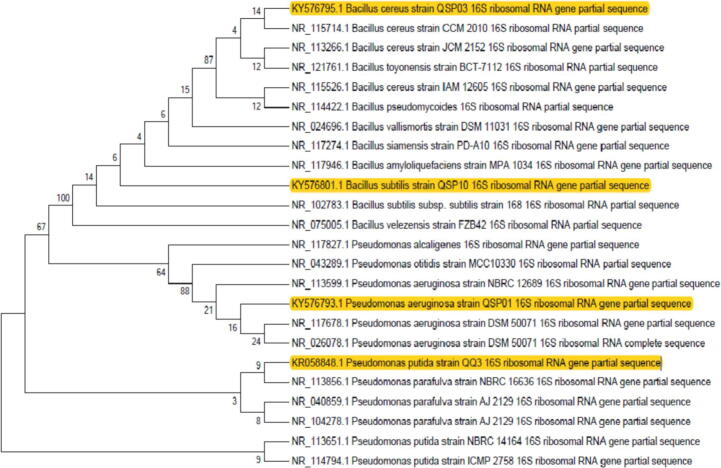
Phylogenetic analysis of the bacterial isolates showing evolutionary relationship. The phylogenetic tree was constructed in Mega 7 by neighbor joining method with a bootstrap value of 100. QQ Bacterial isolates of this study cladded with Bacillus and Pseudomonas sp.

Phylogenetic analysis showed that QQ isolates belonged to the species *Bacillus* and *Pseudomonas*. Isolates QSP01 and QQ3 belonged to the genus *Pseudomonas*., and isolates QSP03 and QSP10 belonged to *Bacillus* sp. Based on 16S rRNA gene sequence homology; isolate QSP01 showed 99% similarity to *P. aeruginosa* NBRC 12689 and submitted under the accession number KY576793. Isolate QSP03 showed 99% similarity to *Bacillus cereus* CCM2010 and given the accession number KY576795. Similarly, QSP10 showed 99% similarity to *Bacillus subtilis* 168 and was given the accession number KY576801. Isolate QQ3 showed 99% similarity to *Pseudomonas putida* KT2440 and given the accession number KR058848. The phylogenetic relationship of the QQ isolates discussed in this study and other closely related bacteria is shown in Fig. 5. Bacterial isolates of this study cladded with *Bacillus* and *Pseudomonas*.

### 3.4 Selected QQ strains showed sensitivity towards antibiotics

**Fig. 6:**
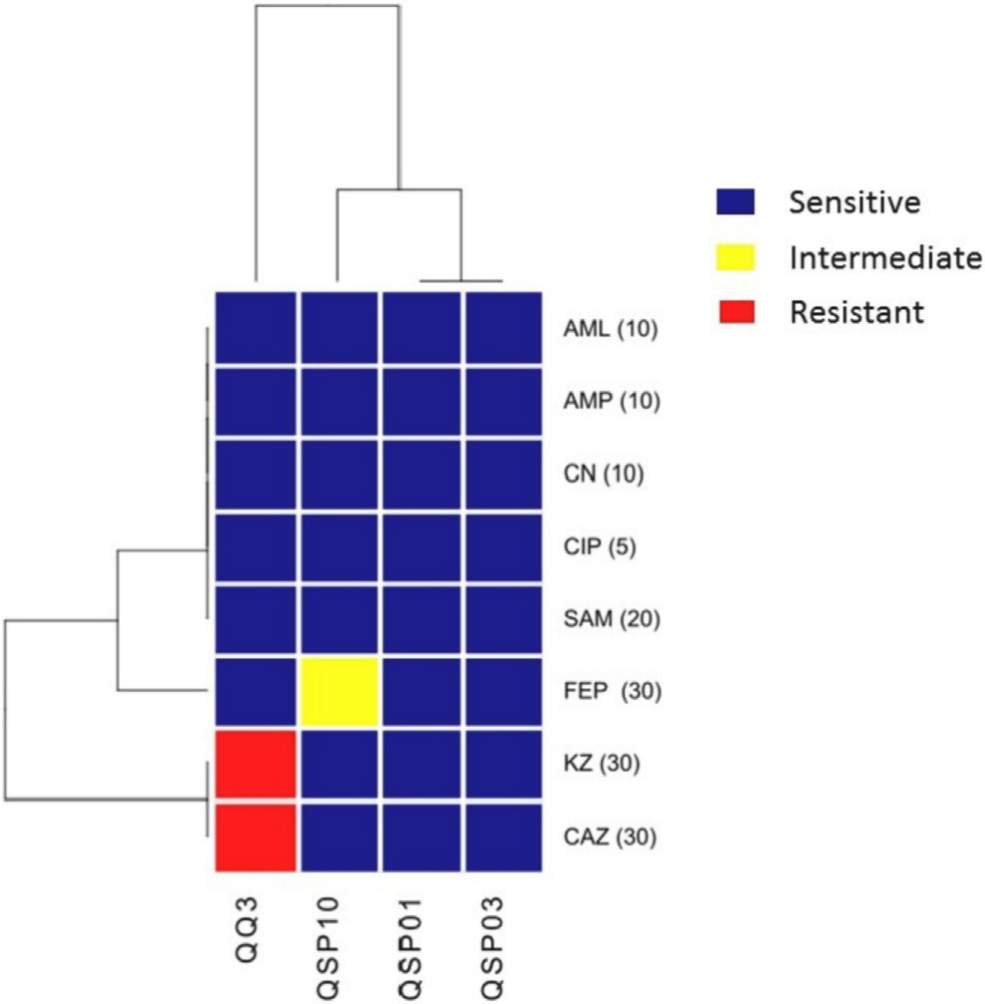
Heat map depicting each QQ isolate as a column and each row as an antibiotic.

Susceptibility test results showed that QQ isolates were generally sensitive to the tested antibiotics. Strains QSP01 and QSP03 were sensitive to all the tested antibiotics. Strain QSP10 was sensitive to all antibiotics except cefepime (FEP, 30 μg) against which it showed intermediate sensitivity. Strain QQ3 showed resistance against cephazolin (KZ, 30 μg), and ceftazidime (CAZ, 30 μg) (Fig. 6). The diameters of zones of inhibition were measured in millimeters and evaluated as Susceptible (S), Intermediate (I), or Resistant (R) according to the CLSI (CLSI, 2012). The interpretation of antibiotic susceptibility was subjected to generate a heat map depicting the hierarchical clustering of each isolate in g-plot package of R (R studio).

### 3.5 Genes encoding lactonases and acylases enzymes are present in all the isolates

**Fig. 7:**
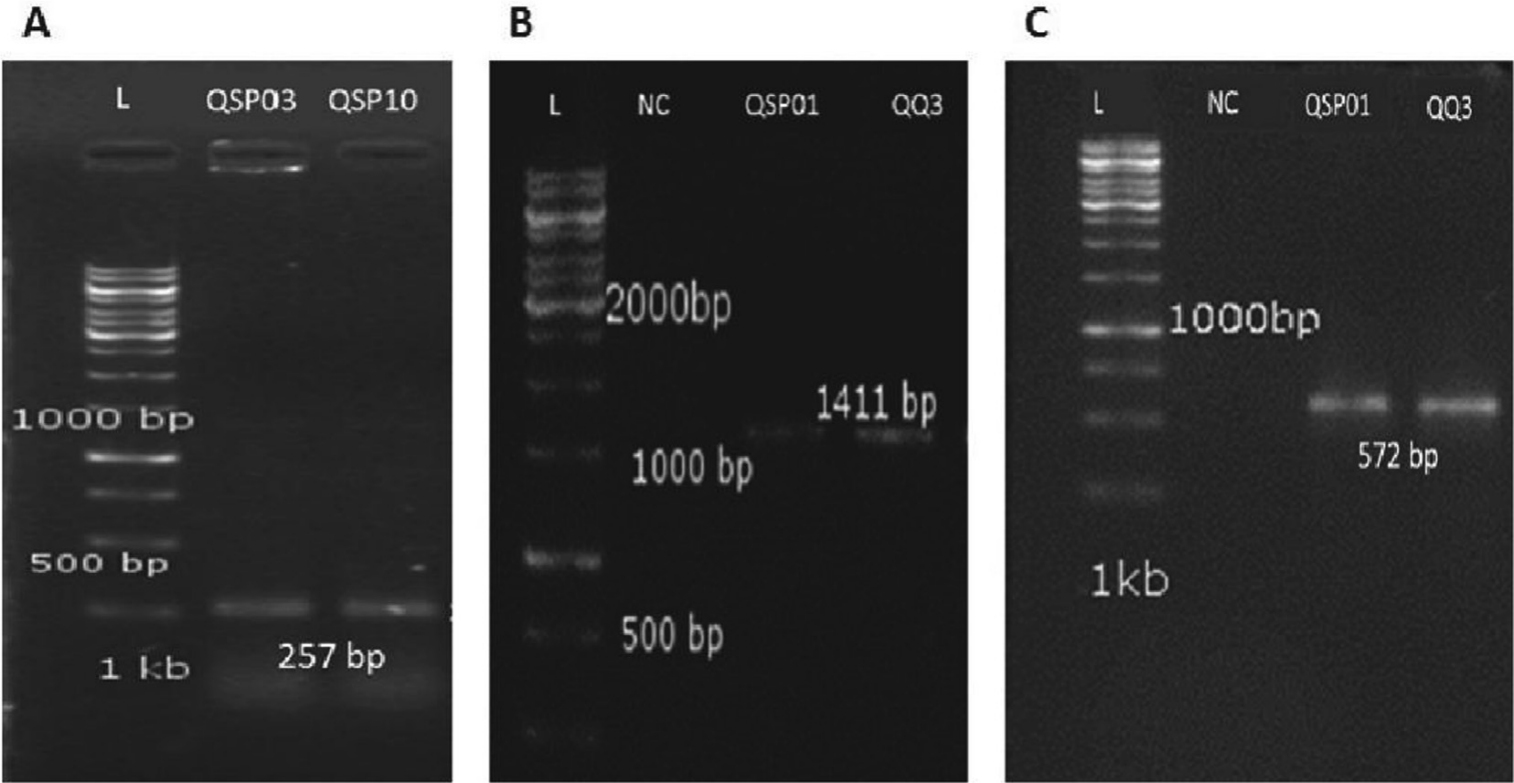
Agarose gel images showing QQ genes amplification through PCR. (A) 257 bp amplified sequence of aiiA gene in Bacillus cereus strain QSP03, and Bacillus subtilis strain QSP10 (B) 1411 bp amplified sequence of pvdQ gene in Pseudomonas aeruginosa strain QSP01, and Pseudomonas putida strain QQ3 (C) 572 bp amplified sequence of quiP gene in Pseudomonas aeruginosa strain QSP01, and Pseudomonas putida strain QQ3.

**Fig. 8:**
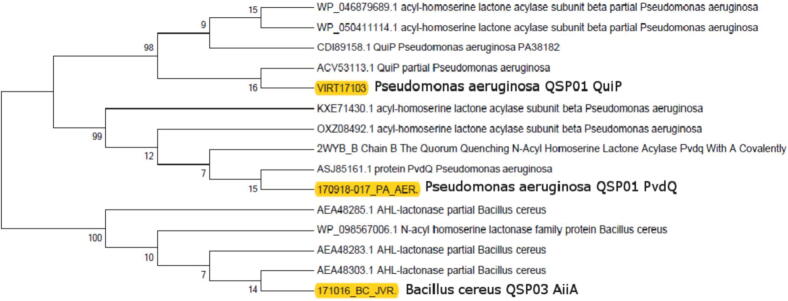
For evolutionary analysis of the bacterial QQ genes, an unrooted phylogenetic tree was constructed in Mega 7 by neighbor joining method with a bootstrap value of 100. It showed that pvdO and quiP genes are homologous and closely related and both belonging to acylase family, whereas AHL lactonase is different belonging to lactonase family.

Already reported literature confirms the presence of AHL lactonase in gram positive bacteria and AHL acylase in gram negative bacteria. Both of these enzymes are reported for Quorum Quenching. Genes *quiP* (572 bp) and *pvdO* (1141 bp) encoding QQ enzyme AHL acylase were amplified in QSP01 and QQ3, both of which are Gram-negative bacteria. Similarly, *aiiA* (257 bp) gene encoding AHL lactonase was amplified in QSP03 and QSP10, both of them are Gram-positive bacteria (Fig. 7)**.** For the sequence analysis of QQ genes, amino acid sequences were retrieved from online available tool ExPASy and for further comparison with already published data, those sequences were subjected to BLAST which showed that AHL lactonase sequence of *Bacillus cereus* QSP03 showed significant alignment with AHL lactonase sequence of *Bacillus cereus* already submitted in NCBI.CDD analysis showed that the AHL lactonase belong to the Metallo-Hydrolase like MBL-fold superfamily of proteins. More analysis showed that Metallo-β-lactamase and metal dependent hydrolase were the two conserved domains found in AHL lactonase sequence. Sequenced amplicons of *pvdO* and *quiP*, both encode for AHL acylase, showed high similarity with AHL acylase sequence of *P*. *aeruginosa* already submitted in NCBI. CDD analysis further showed that *pvdO* and *quiP* belong to Ntn-hydrolase superfamily and penicillin-amidase superfamily of proteins, respectively. An unrooted phylogenetic tree was generated with neighbor joining method that showed *pvdO* and *quiP* genes are homologous and closely related and both belonging to acylase family, whereas AHL lactonase is different belonging to lactonase family (Fig. 8).

## 4 Discussion

Bacteria are evolving continuously against antibiotics which has decreased the efficiency of antibiotic treatment against them (Beceiro et al., 2013). Antibiotic resistance is not a problem only in healthcare sector, in fact membrane biofouling in wastewater treatment plant is also due to microorganisms forming biofilms and all the available treatments fail to inhibit the formation of natural biofilms (Oh et al., 2013). Main purpose of the study was to find out new quorum quenching bacterial strains and detect molecular basis of their QQ activity as it‘s an innovative strategy to combat the increasing challenges posed by multi-drug resistant bacteria and their biofilms in the healthcare sector.

Autoinducers play a role in quorum sensing and hence biofilm formation. AI concentration increases with bacterial community density. Receptors for the detection of these signaling molecules are present in bacterial membrane or cytoplasm. The loop known as “feed-forward AI’s loop” detects AI concentration and activates modifications in gene expression which in turn leads to increment in AI’s secretion. Two-component histidine kinase receptor present in membrane, serve as receptor for these signaling molecules. A series of reaction is initiated first by autophosphorylation of regulator in cytoplasm and then by activation of transcription of bacterial gene by activated regulator. However, this mechanism differs in types of signaling molecules i.e. AI-1 and AI-2. AI-1 molecules involve acyl-homoserine lactones and it controls intra-specific communication. Whereas, AI-2 involves furanosyl borate diester (Samrot et al., 2021)

The mechanism also differ depending upon the type of bacteria. In Gram positive bacteria, small molecules called auto-inducing peptides (AIPs) are produced. The gram positive bacteria regulate gene expression by making use of signaling molecules called oligopeptides which are small post-translationally processed peptide signaling molecules. These signaling molecules are secreted and then are either reinternalized via an oligopeptide transport system or detected at the bacterial surface by two-component systems. LuxI/ LuxR type Quorum Sensing system exists in Gram negative bacteria (Hastings and Greenberg 1999). The main Gram Negative bacterial species belonging to proteobacteria contain AHL- mediated Quorum Sensing system (Hardman et al., 1998). AHLs are primary signaling molecules in Gram negative bacteria. They are small, neutral lipids and amphipathic in nature. AHL synthesis has been discovered in about 90 species of bacteria. Total three enzymes have been reported yet to produce AHLs. They belong to LuxM, HdtS and LuxI families (Water and Bassler, 2005). The typical example of LuxI type AHL synthase was first described in *Vibrio fischeri.* S-adenosyl Methionine (SAM) and acyl-acyl Carrier protein (acyl-ACP) serve as substrate for production of AHLs in LuxI type AHL synthase (Hanzelka and Greenberg 1996). In Vitro studies have confirmed the requirement of SAM and acyl-ACP substrates for AHL production (More and Finger, 1996).

Therefore, quorum sensing is an important process in bacteria and diligently involved in regulation of virulent phenotypes, it is a potential target for a novel therapeutic approach as it has roles in bacterial infection as well as in biofilm formation. One such approach is the isolation and characterization of QQ bacteria due to its obvious potential benefits. In this study, QQ bacteria were isolated from the sludge of membrane bioreactor and their QQ activity was confirmed through various AHL degradation assays. Many QQ and QS bacteria have been reported in terrestrial as well as aquatic environments (Tan et al., 2015, Saurav et al., 2016). For this study, sludge from the membrane bioreactor was used because this particular niche has not been explored much from the point of view of QQ bacteria. Moreover, this niche might help in explore novel bacterial species that can be used for biofilm control not only in health sector but to also control biofouling on membrane bioreactors (MBR).

For this study, QQ bacteria were isolated on selective media supplemented with AHLs as sole source of carbon and nitrogen (Dong et al., 2000, Christiaen et al., 2011). Only four QQ isolates were selected for further studies, selection was done on the basis of growth on selective media. The selected strains showed AHL degradation capability when tested with biosensor strains CV026 and A136 which respond to exogenous AHLs by pigment production. Isolated bacterial strains significantly inhibited the pigment production by degrading AHLs provided from exogenous source (McClean et al., 1997).

To quantitate the AHL degradation by our isolated bacterial strains, AHL degradation assay by microtiter plate method was performed, using a biosensor strain CV026 which responds to exogenous AHLs by producing violacein. Current QQ bacterial strains were positive for AHL degradation to different extents as measured by intensity of violacein produced (Cady et al., 2012, Rajesh and Ravishankar Rai, 2014). It is already proved in previous studies that QQ bacteria can effectively degrade small chain AHLs compared to the long chain AHLs (Romero et al., 2014, Torres et al., 2013, Tan et al., 2015). This suggests that in future, the search of QQ isolates can only be dependent of their capability to degrade small chain AHLs.

*Pseudomonas aeruginosa* is an opportunistic pathogen and involved in biofilm associated complicated infections and multidrug resistance. Its Quorum Sensing mechanism has been extensively characterized and a complete QS mechanism is required in *P*. *aeruginosa* to be virulent and any disruption in this mechanism can lead to its decreased pathogenicity (Rasamiravaka et al., 2015, Sio et al., 2006) as well as deficiency in biofilm formation (Shih and Huang, 2002). Quorum quenching enzymes AHL lactonase and AHL acylase have the ability to suppress biofilm formation in P*seudomonas* sp. (Dong et al., 2002). Inhibitory effect of cell free lysates of QQ isolates was tested against *P*. *aeruginosa* biofilms. QQ isolates showed significant inhibition of biofilm formation in *P. aeruginosa* (Rajesh and Ravishankar Rai, 2014)*.* To confirm the fact that biofilm inhibition ability of our isolates is due to activity of QQ enzymes in their cell free lysates, exogenous C4-HSL was added along with cell free lysates. Its addition caused regeneration of biofilms in treated cells more significantly in QSP03 and QQ3 strains. This demonstrates the role of AHLs and quorum sensing in *P*. *aeruginosa* biofilm formation. C4-HSL is a quorum sensing molecule and plays a significant role in biofilm formation of *P. aeruginosa* (Favre-Bonte et al., 2003). Planktonic cells were not affected in this study which shows that cell free lysate only target QS by AHL degradation instead of whole biomass (Rajesh and Ravishankar Rai, 2014). Defects in AHL producing mechanism or inability to produce AHL reported to be the cause of lack of pathogenicity in some plant pathogens (Rashid et al., 2011). Hence, AHL degradation could be a potential strategy to suppress the pathogenicity and decrease in the severity of disease.

Quorum Quenching strains were tested for their antibiotic susceptibility as the antibiotic resistance is linked with pathogenic potential. Some reports also suggest the link between higher virulence and higher resistance in microorganisms (Beceiro et al., 2013). Study of infections in animal models have suggested a direct link between virulence and antibiotic resistance (Roux et al., 2015). The tested QQ strains were generally susceptible to all the tested antibiotics; only strain QQ3 showed resistance against cephazolin (KZ, 30 μg), and ceftazidime (CAZ, 30 μg). So, the tested QQ isolates were generally regarded as non-pathogens on the basis of their susceptibility.

AHL lactonase and acylase genes were amplified for identification of enzymes responsible for AHL degradation activity in QQ strains. Three gene including *aiiA* encoding AHL lactonase in *B. cereus* QSP03 and *pvdQ* and *quiP* encoding AHL acylase in *P. aeruginosa* QSP01 were sequenced (Sio et al., 2006, Dong et al., 2002).

Amino acid sequence of *aiiA* amplicon was determined by online ExPASy tool, that was significantly identical to AHL lactonase sequences of *Bacillus cereus* in NCBI database. Metallo- β lactamase and metal dependent hydrolase were identified as two conserved domains in ExPASy retrieved AHL lactonase amino acid sequence of bacterial isolate *B. cereus* QSP03 through CDD analysis. According to a reported work (Dong et al., 2002) AHL lactonase in *B*. *cereus* contained two conserved region which were Zinc containing glyoxylase and Metallo-Beta-lactamase II. These two conserved regions contain several glutamate and histidine residues required for binding of zinc and optimal enzyme activity (Crowder et al., 1997). So, the presence of these two domains i.e. Metallo- β -lactamase and metal dependent hydrolase in our AHL lactonase sequence shows that these AHL degrading enzymes also have conserved zinc binding motif which is important for catalytic activity of enzyme. Moreover, CDD analysis also showed the relatedness of AHL lactonase amino acid sequence of *B. cereus* QSP03 to Metallo-Hydrolase-like_MBL-fold superfamily. This superfamily of proteins specifically involves hydrolytic enzymes including AHL lactonase, beta-lactamases, and persulfide dioxygenase performing the number of biological functions (Marchler-Bauer et al., 2017).

Retrieved amino acid sequence of AHL acylase for both *pvdQ* and *quiP* also showed high similarity to already published sequences of the AHL acylase encoding genes in NCBI. CDD analysis showed both belong to Ntn-hydrolase superfamily and penicil-amidase superfamily of proteins, respectively. *pvdQ* encoding enzyme AHL acylase is an Ntn-hydrolase (Bokhove et al., 2010) and well reported for its QQ activity (Papaioannou et al., 2009). *quiP*, an Ntn-hydrolase is also present in P. *aeruginosa* genome and exhibit same specificity for substrate as PvdQ (Huang et al., 2006).

## 5 Conclusion

The isolated strains from sludge of membrane bioreactor, were capable to degrade AHLs of various acyl lengths. Phylogenetic analysis of strains showed that the isolated QQ strains belong to the species of *Bacillus* and *Pseudomonas*. These strains successfully inhibited the biofilm formation of *P. aeruginosa* strain by around 60%. The ability of our bacterial isolates to quench the quorum sensing and identification of AHL lactonase and AHL acylase which are two main enzymes involved in AHL degradation provides a new source of these enzymes. Degradation of *P*. *aeruginosa* biofilms *in vitro* by these bacterial isolates could be feasible in the clinical situations too.

## Declaration of Competing Interest

The authors declare that they have no known competing financial interests or personal relationships that could have appeared to influence the work reported in this paper.

## References

[b0005] Abdula N., Macharia J., Motsoaledi A., Swaminathan S., Vijay Raghavan K. (2016). National action for global gains in antimicrobial resistance. Lancet.

[b0010] Adonizio A., Kong K.F. (2008). Inhibition of quorum sensing-controlled virulence factor production in *Pseudomonas aeruginosa* by South Florida plant extracts. Antimicrob. Agents Chemother..

[b0015] Barry A.L., Thornsberry, C., 1985. In: Lennette, E.H., Hausler Jr., W.J., Shadomy, H.J. (Eds.), Susceptibility tests: Diffusion test procedures. Am Soc Microbiol. 4th ed. Washington, D.C, pp. 978–987.

[b0020] Beceiro A., Tomas M. (2013). Antimicrobial resistance and virulence: a successful or deleterious association in the bacterial world?. Clin. Microbiol. Rev..

[b0025] Bokhove M., Nadal Jimenez P. (2010). The quorum-quenching N-acyl homoserine lactone acylase *pvdQ* is an Ntn-hydrolase with an unusual substrate-binding pocket. Proc. Natl. Acad. Sci. USA.

[b0030] Bzdrenga J., Daude D., Remy B., Jacquet P., Plener L., Elias M. (2017). Biotechnological applications of quorum quenching enzymes. Chem. Biol. Interact..

[b0035] Cady N.C., McKean K.A. (2012). Inhibition of biofilm formation, quorum sensing and infection in *Pseudomonas aeruginosa* by natural products-inspired organosulfur compounds. PLoS ONE.

[b0040] Chan K.G., Atkinson S. (2011). Characterization of N-acylhomoserine lactone-degrading bacteria associated with the *Zingiber officinale* (ginger) rhizosphere: co-existence of quorum quenching and quorum sensing in *Acinetobacter* and *Burkholderia*. BMC Microbiol..

[b0045] Chen F., Gao Y. (2013). Quorum quenching enzymes and their application in degrading signal molecules to block quorum sensing-dependent infection. Int. J. Mol. Sci..

[b0050] Chioro A., Coll-Seck A.M., Hoie B., Moeloek N., Motsoaledi A., Rajatanavin R. (2015). & Touraine, M. Antimicrobial resistance: A priority for global health action. Bull..

[b0055] Chong T.M., Koh C.L. (2012). Characterization of quorum sensing and quorum quenching soil bacteria isolated from Malaysian tropical montane forest. Sensors Basel).

[b0060] Chowdhary P.K., Keshavan N., Nguyen H.Q., Peterson J.A., Gonzalez J.E., Haines D.C. (2007). *Bacillus megaterium* CYP102A1 oxidation of acylhomoserine lactones and acyl homoserines. Biochemistry.

[b0065] Christiaen S.E., Brackman G. (2011). Isolation and identification of quorum quenching bacteria from environmental samples. J. Microbiol. Methods.

[b0070] Clinical and Laboratory Standards Institute (CLSI), 2012. Performance standards for antimicrobial susceptibility testing. Twenty-second Informational Supplement CLSI Document M100-S22.

[b0075] Crowder M.W., Maiti M.K. (1997). Glyoxalase II from A. thaliana requires Zn(II) for catalytic activity. FEBS Lett..

[b0080] Dong Y.H., Zhang L.H. (2005). Quorum sensing and quorum-quenching enzymes. J. Microbiol..

[b0085] Dong Y.H., Gusti A.R. (2002). Identification of quorum-quenching N-acyl homoserine lactonases from *Bacillus* species. Appl. Environ. Microbiol..

[b0090] Dong Y.H., Xu J.L. (2000). *aiiA*, an enzyme that inactivates the acylhomoserine lactone quorum-sensing signal and attenuates the virulence of *Erwinia carotovora*. PNAS.

[b0095] Favre-Bonte S., Kohler T. (2003). Biofilm formation by *Pseudomonas aeruginosa*: role of the C4-HSL cell-to-cell signal and inhibition by azithromycin. J. Antimicrob. Chemother..

[b0100] Galloway W.R., Hodgkinson J.T., Bowden S.D., Welch M., Spring D.R. (2011). Quorum sensing in Gram-negative bacteria: small-molecule modulation of AHL and AI-2 quorum sensing pathways. Chem. Rev..

[b0105] Gao M., Teplitski M. (2003). Production of substances by *Medicago truncatula* that affect bacterial quorum sensing. Mol. Plant Microbe Interact..

[b0110] Haque S., Ahmad F., Dar S.A., Jawed A., Mandal R.K., Wahid M., Lohani M., Khan S., Singh V., Akhter N. (2018). Developments in strategies for Quorum Sensing virulence factor inhibition to combat bacterial drug resistance. Microb. Pathog..

[b0115] Hentzer M., Givskov M. (2003). Pharmacological inhibition of quorum sensing for the treatment of chronic bacterial infections. J. Clin. Invest..

[b0120] Huang J.H., Shi Y.H., Zeng G.M., Gu Y.L., Chen G.Q., Shi L.X. (2016). Acyl-homoserine lactone-based quorum sensing and quorum quenching hold promise to determine the performance of biological wastewater treatments: an overview. Chemosphere.

[b0125] Huang J.J., Petersen A. (2006). Identification of *quiP*, the product of gene PA1032, as the second acyl-homoserine lactone acylase of *Pseudomonas aeruginosa* PAO1. Appl. Environ. Microbiol..

[b0130] Jiang Q., Chen J., Yang C., Yin Y., Yao K. (2019). Quorum Sensing: A prospective therapeutic target for bacterial diseases. BioMed Res. Int..

[b0135] Lade H., Paul D. (2014). Isolation and molecular characterization of biofouling bacteria and profiling of quorum sensing signal molecules from membrane bioreactor activated sludge. Int. J. Mol. Sci..

[b0140] Laxminarayan R., Duse A., Wattal C., Zaidi A.K.M., Wertheim H.F.L., Sumpradit N., Vlieghe E., Levy Hara G., Gould I.M., Goossens H. (2013). Antibiotic resistance-the need for global solutions. Lancet Infect. Dis..

[b0145] Leadbetter J.R., Greenberg E.P. (2000). Metabolism of acyl-homoserine lactone quorum-sensing signals by *Variovorax paradoxus*. J. Bacteriol..

[b0150] Liao X., Ma Y., Daliri E.B.M., Koseki S., Wei S., Liu D., Ye X., Chen S., Ding T. (2019). Interplay of antibiotic resistance and food-associated stress tolerance in foodborne pathogens. Trends. Food Sci. Technol..

[b0155] Lin Y.H., Xu J.L. (2003). Acyl-homoserine lactone acylase from *Ralstonia* strain XJ12B represents a novel and potent class of quorum-quenching enzymes. Mol. Microbiol..

[b0160] Ma Y., Lan G., Li C., Cambaza E.M., Liu D., Ye X., Chen S., Ding T. (2019). Stress tolerance of *Staphylococcus aureus* with different antibiotic resistance profiles. Microb. Pathog..

[b0165] Manefield M., de Nys R. (1999). Evidence that halogenated furanones from *Delisea pulchra* inhibit acylated homoserine lactone (AHL)-mediated gene expression by displacing the AHL signal from its receptor protein. Microbiology.

[b0170] Marchler-Bauer A., Bo Y. (2017). CDD/SPARCLE: functional classification of proteins via subfamily domain architectures. Nucleic Acids Res..

[b0175] McClean K.H., Winson M.K., Fish L., Taylor A., Chhabra S.R., Camara M. (1997). Quorum sensing and *Chromobacterium violaceum*: exploitation of violacein production and inhibition for the detection of N-acylhomoserine lactones. Microbiology.

[b0180] Musk D.J., Hergenrother P.J. (2006). Chemical countermeasures for the control of bacterial biofilms: effective compounds and promising targets. Curr. Med. Chem..

[b0185] O’Niel J. (2016).

[b0190] Oh H.S., Kim S.R. (2013). Biofouling inhibition in MBR by *Rhodococcus* sp. BH4 isolated from real MBR plant. Appl. Microbiol. Biotechnol..

[b0195] Ozer E.A., Pezzulo A. (2005). Human and murine paraoxonase 1 are host modulators of *Pseudomonas aeruginosa* quorum-sensing. FEMS Microbiol. Lett..

[b0200] Papaioannou E., Wahjudi M. (2009). Quorum-quenching acylase reduces the virulence of *Pseudomonas aeruginosa* in a Caenorhabditis elegans infection model. Antimicrob. Agents Chemother..

[b0205] Rajesh P.S., Ravishankar Rai V. (2014). Quorum quenching activity in cell-free lysate of endophytic bacteria isolated from *Pterocarpus santalinus* Linn., and its effect on quorum sensing regulated biofilm in *Pseudomonas aeruginosa* PAO1. Microbiol. Res..

[b0210] Rasamiravaka T., Vandeputte O.M. (2015). *Pseudomonas aeruginosa* Biofilm Formation and Persistence, along with the Production of Quorum Sensing-Dependent Virulence Factors, Are Disrupted by a Triterpenoid Coumarate Ester Isolated from *Dalbergia trichocarpa*, a Tropical Legume. PLoS ONE.

[b0215] Rashid R., Morohoshi T. (2011). Degradation of N-acylhomoserine lactone quorum sensing signaling molecules by potato root surface-associated *Chryseobacterium* strains. Microbes Environ..

[b0220] Rasmussen T.B., Givskov M. (2006). Quorum-sensing inhibitors as anti-pathogenic drugs. Int. J. Med. Microbiol..

[b0225] Reverchon S., Chantegrel B. (2002). New synthetic analogues of N-acyl homoserine lactones as agonists or antagonists of transcriptional regulators involved in bacterial quorum sensing. Bioorg. Med. Chem. Lett..

[b0230] Romero M., Muras A., Mayer C., Bujan N., Magarinos B., Otero A. (2014). *In vitro* quenching of fish pathogen *Edwardsiella tarda* AHL production using marine bacterium *Tenacibaculum* sp. strain 20J cell extracts. Dis. Aquat. Organ..

[b0235] Roux D., Danilchanka O. (2015). Fitness cost of antibiotic susceptibility during bacterial infection. Sci. Transl. Med..

[b0240] Saurav K., Bar-Shalom R., Haber M., Burgsdort I., Oliviero G., Costantino V. (2016). In search of alternative antibiotic drugs: quorum-quenching activity in sponges and their bacterial isolates. Front. Microbiol..

[b0245] Shih P.C., Huang C.T. (2002). Effects of quorum-sensing deficiency on *Pseudomonas aeruginosa* biofilm formation and antibiotic resistance. J. Antimicrob. Chemother..

[b0250] Sio C.F., Otten L.G. (2006). Quorum quenching by an N-acyl-homoserine lactone acylase from *Pseudomonas aeruginosa* PAO1. Infect. Immun..

[b0255] Tan C.H., Koh K.S., Xie C., Zhang J., Tan X.H., Lee G.P. (2015). Community quorum sensing signalling and quenching: microbial granular biofilm assembly. Biofilms Microbiomes..

[b0260] Thompson J.D., Higgins D.G., Gibson T.J. (1994). CLUSTAL-W—Improving the sensitivity of progressive multiple sequence alignment through sequence weighting, position-specific gap penalties and weight matrix choice. Nucleic Acids Res..

[b0265] Torres M., Romero M., Prado S., Dubert J., Tahrioui A., Otero A. (2013). N-acylhomoserine lactone-degrading bacteria isolated from hatchery bivalve larval cultures. Microbiol. Res..

[b0270] Torres M., Rubio-Portillo E., Anton J., Ramos-Espla A.A., Quesada E., Llamas I. (2016). Selection of the N-acylhomoserine lactone-degrading bacterium *Alteromonas stellipolaris* PQQ-42 and of its potential for biocontrol in aquaculture. Front. Microbiol..

[b0275] Whitehead N.A., Barnard A.M. (2001). Quorum-sensing in gram-negative bacteria. FEMS Microbiol. Rev..

[b0280] Whiteley M., Diggle S.P., Greenberg E.P. (2017). Progress in and promise of bacterial quorum sensing research. Nature.

[b0285] Williams P., Winzer K., Chan W.C., Camara M. (2007). Look who’s talking: communication and quorum sensing in the bacterial world. Phil. Trans. R. Soc..

[b0290] Withers H., Swift S., Williams P. (2001). Quorum sensing as an integral component of gene regulatory networks in Gram-negative bacteria. Curr. Opin. Microbiol..

[b0295] Zhang L.H., Dong Y.H. (2004). Quorum sensing and signal interference: diverse implications. Mol. Microbiol..

[b0300] Zhao X.H., Zhong J.L., Wei C.J., Lin C.W., Ding T. (2017). Current perspectives on viable but non-culturable state in foodborne pathogens. Front. Microbiol..

[b0305] Zhao X., Zhao F., Wang J., Zhong N. (2017). Biofilm formation and control strategies of foodborne pathogens: Food safety perspectives. RSC Adv..

[b0310] Zhu H., He C.C. (2011). Inhibition of quorum sensing in *Chromobacterium violaceum* by pigments extracted from Auricularia auricular. Lett. Appl. Microbiol..

[b0315] Samrot A.V., Mohamed A.A. (2021). Mechanisms and impact of biofilms and targeting of biofilms using bioactive compounds-a review. Medicina..

[b0320] Hastings J.W., Greenberg E.P. (1999). Quorum sensing: the explanation of a curious phenomenon reveals a common characteristic of bacteria. J. Bacteriol..

[b0325] Hardman A.M., Stewart G.S. (1998). Quorum sensing and the cell-cell communication dependent regulation of gene expression in pathogenic and non-pathogenic bacteria. Antonie Van Leeuwenhoek.

[b0330] Water C.M., Bassler B.L. (2005). Quorum sensing: cell-to-cell communication in bacteria. Annu. Rev. Cell Dev. Biol..

[b0335] Hanzelka B.L., Greenberg E.P. (1996). Quorum sensing in Vibrio fischeri: evidence that S-adenosylmethionine is the amino acid substrate for autoinducer synthesis. J. Bacteriol..

[b0340] More M.I., Finger L.D. (1996). Enzymatic synthesis of a quorum-sensing autoinducer through use of defined substrates. Science.

